# Upregulation of miR-135b Is Involved in the Impaired Osteogenic Differentiation of Mesenchymal Stem Cells Derived from Multiple Myeloma Patients

**DOI:** 10.1371/journal.pone.0079752

**Published:** 2013-11-06

**Authors:** Song Xu, Gaia Cecilia Santini, Kim De Veirman, Isabelle Vande Broek, Xavier Leleu, Ann De Becker, Ben Van Camp, Karin Vanderkerken, Ivan Van Riet

**Affiliations:** 1 Department of Lung Cancer Surgery, Lung Cancer Institute, Tianjin Medical University General Hospital, Tianjin, P.R.China; 2 Stem Cell Laboratory-Division Clinical Hematology, Universitair Ziekenhuis Brussel (UZ Brussel), Brussels, Belgium; 3 Department of Hematology and Immunology-Vrije Universiteit Brussel (VUB), Myeloma Center Brussels, Brussels, Belgium; 4 Service d'Hématologie, Centre Hospitalier Universitaire (CHU), Lille, France; Rutgers - New Jersey Medical School, United States of America

## Abstract

Previous studies have demonstrated that mesenchymal stem cells from multiple myeloma (MM) patients (MM-hMSCs) display a distinctive gene expression profile, an enhanced production of cytokines and an impaired osteogenic differentiation ability compared to normal donors (ND-hMSCs). However, the underlying molecular mechanisms are unclear. In the present study, we observed that MM-hMSCs exhibited an abnormal upregulation of miR-135b, showing meanwhile an impaired osteogenic differentiation and a decrease of SMAD5 expression, which is the target of miR-135b involved in osteogenesis. By gain and loss of function studies we confirmed that miR-135b negatively regulated hMSCs osteogenesis. We also found that MM cell-produced factors stimulated ND-hMSCs to upregulate the expression of miR-135b. Importantly, treatment with a miR-135b inhibitor promoted osteogenic differentiation in MM-hMSCs. Finally, we observed that MM cell-derived soluble factors could induce an upregulation of miR-135b expression in ND-hMSCs in an indirect coculture system and the miR-135b expression turned to normal level after the removal of MM cells. Collectively, we provide evidence that miR-135b is involved in the impaired osteogenic differentiation of MSCs derived from MM patients and might therefore be a promising target for controlling bone disease.

## Introduction

Multiple myeloma (MM) is a hematological malignancy characterized by clonal proliferation of plasma cells in the bone marrow (BM). This disease is associated with several clinical manifestations but the major hallmark is the occurrence of bone fractures, prominently located in the central skeleton, skull and long bones. Bone lesions are caused by the dysregulation of bone homeostasis with an abnormal osteoclast activation and osteoblast inhibition within the MM BM microenvironment. 

Several groups have reported that BM-derived mesenchymal stem cells from MM patients (MM-hMSCs) show a distinctive gene expression profile and an enhanced production of cytokines, including IL-6, DKK1, IL-1β, and SDF-1 [1-4]. Moreover, we demonstrated that MM-hMSCs also have an impaired osteogenic differentiation ability, compared to normal donor-derived hMSCs (ND-hMSCs) [5]. However, the molecular mechanisms associated with these abnormalities in MM-hMSCs are not understood well.

MicroRNAs (miRNAs) are endogenous non-coding RNA molecules, which have an important regulatory role in gene expression. miRNAs are involved in many biological processes and their aberrant expression can lead to cancer formation and progression. Recent studies have illustrated that the miRNA expression pattern in MM is associated with genetic abnormalities and identified several specific miRNAs which can regulate critical genes associated with MM pathogenesis [6,7]. Importantly, miRNAs play a crucial role in regulating stem cells fate and are also involved in the differentiation process of mesenchymal stem cells (MSCs) [8,9]. Previous studies have demonstrated that a microRNA signature was associated with osteogenic lineage commitment, and some microRNAs, including miR-142-3p, miR-100, miR-135, miR-155, miR-34c, miR-182, miR-22 and others, have been proven to be functionally involved in the regulation of osteogenic differentiation by targeting bone formation related pathways components [10-16]. In the present study, we provide evidence that the abnormal miRNA expression is related to the impaired osteogenic differentiation ability of MM-hMSCs.

## Materials and Methods

### Ethics Statement

The study was conducted in accordance with the Helsinki Declaration. Patient samples have been collected with the approval of the Ethics Board of the University Hospital Brussels UZ Brussel (BUN14320097462). Healthy donors provided written informed consent and myeloma patients provided verbal informed consent. The reason that written consent was not obtained for myeloma patients is: The patient samples that were used in this study were considered as “waste samples”. According to Belgian legislation, a formal informed consent is not required to use this type of samples. When waste samples are used for scientific research, the consent is assumed to be given unless the patient specifically indicates otherwise before the sample are collected. Patients at our institution are aware of this right to refuse the use of the samples. All patient information was kept strictly confidential. The ethics committees approve this consent procedure.

### Primary culture of human MSCs

Bone marrow samples of myeloma patients and healthy donors were obtained after verbal and written informed consent. Bone marrow aspirates were obtained from sternum of healthy donors, or from the iliac crest of myeloma patients. For hMSCs from healthy donors, BM mononuclear cells were isolated by density gradient centrifugation with Ficoll-Hypaque (Nycomed, Lucron Bioproducts, De Pinte, Belgium), and the mononuclear cell fraction was collected and washed in phosphate-buffered saline (PBS) (Gibco, Invitrogen, Merelbeke Belgium). The number of mononuclear cells was counted by Turck staining. For primary culturing, 20 million cells were seeded in T-25 Nunclon culture flasks (Nunc, VWR International Leuven, Belgium) in 5mL MesenPro medium (Invitrogen) containing 2% fetal calf serum (FCS), 1% antibiotic/antimycotic (penicillin 10.000 U/ml; streptomycin 10mg/ml), 1% L-glutamine and 2% MesenPro growth supplement (Invitrogen). After 24h, non-adherent cells were discarded by PBS wash, and adherent cells were cultured at 37°C in 5% humidified CO_2_. Medium was refreshed every 3-4 days until 80-90% confluence was reached. After about 7-10 days, the cells were detached with 0.25% trypsin/0.02% EDTA. After trypsin neutralization, the cell suspension was centrifuged at room temperature for 5 minutes at 1200 rpm, the supernatant was removed and the cells were resuspended, counted with trypan blue staining and plated into new flasks at 2000 cells/ cm^2^. Subsequent passages were performed similarly, but split ratios were 1:2 (T-75 flask). For hMSCs from MM patients, an additional CD138 MACS separation step was performed to remove the malignant plasma cells before the initial culture. hMSCs were used at passage 2 in this study. Prior to use in experiments, hMSCs were characterized by their fibroblast-like morphology, immunophenotype (CD90+, CD73+, CD166+, CD105+ and CD45-) and differentiation ability towards adipocytes, osteoblasts and chondrocytes in specific induction media. 

### miRNA array analysis

Total RNA, including small RNAs, from ND-hMSC and MM-hMSC was extracted using the miReasy® Mini kit (Qiagen, Westburg, Leusden, Netherlands). High-throughput miRNA expression profiling was performed by service provider Biogazelle (Ghent, Belgium). A validated miRNA screening pipeline was used that allowed for accurate and sensitive expression analysis of 755 microRNAs by means of real-time quantitative PCR with hydrolysis probe based miRNA assays [17]. In brief, 60 ng of total RNA was reverse transcribed using the Megaplex RT stem-loop primer pool (Applied Biosystems), enabling miRNA specific cDNA synthesis of 755 different human miRNAs and small RNA controls. Subsequently, the Megaplex RT product was pre-amplified by means of a 12-cycle PCR reaction with a miRNA specific forward primer and universal reverse primer to increase detection sensitivity. Finally, a 1,600-fold dilution of pre-amplified miRNA cDNA was used as input for a 40-cycle qPCR reaction with miRNA specific hydrolysis probes and primers (Applied Biosystems). All measurements were performed in 384-well plates in a reaction volume of 8 μl (7900 HT, Applied Biosystems) using the gene maximization strategy. The Cq values were subsequently analyzed in qbase^PLUS^ [18] following an improved version of the global mean normalization procedure described by Mestdagh et al. [19]. Expression alteration of 1.5-fold was defined as threshold difference according to the company suggestions and previous reports [19]. 

### Luciferase reporter assay

For the luciferase reporter assay, 3’UTR fragments of SMAD5, containing the putative binding sites for miR-135b, were subcloned into pmiR-RB-REPORTTM luciferase reporter vectors (RiboBio Co Ltd, Guangzhou, China.). The 3′UTR sequences were amplified from human cDNA by PCR using the following primers: F: 5' CCGCTCGAGTGAAAGGAAAGTACCTCTGAAAG 3', R: 5' GAATGCGGCCGCTCTTAAGAGTTATGGCTTTC 3'. Mutant 3’UTR of SMAD5 was constructed, carrying mutated sequences in the complementary sites for the seed region of miR-135b, and inserted into the pmiR-RB-REPORTTM control vector at the same sites. For reporter assays, 1.5×10^4^ HEK 293 cells were seeded into 96-well plates. Once the cells had reached 60–70% confluence, they were cotransfected with wild-type (or mutant) reporter plasmid and miR-135b mimic (or negative control) using Lipofectamine 2000 (Invitrogen). The activity of firefly and Renilla luciferase were measured sequentially in cell lysates 48h post-transfection using the Dual-Luciferase Reporter Assay system according to the manufacturer’s instructions (Promega, Madison, WI, USA).

### Quantitative real time PCR

#### qPCR for miR-135b

Total RNA, including small RNAs, was extracted using the miReasy^®^ Mini kit (Qiagen) and reverse transcription for miRNA was performed with miScript Reverse Transcription Kit (Qiagen) according to the manufacturer’s protocol. qPCR for microRNA was performed by miScript SYBR Green PCR Kit (Qiagen) with the primer of miR-135b (MS00003472, Qiagen), using iCycler Thermal Cycler (Biorad, Nazareth, Belgium). miR-135b expression were normalised to the endogenous U6(MS00014000, Qiagen).

#### qPCR for osteogenic markers

Total RNA was extracted with Trizol reagent (Invitrogen, Merelbeke Belgium) and Rneasy^®^ Mini kit (Qiagen), and cDNA was synthesized using Thermo Scientific Verso^TM^ cDNA synthesis kit (Thermo Scientific ABgene, Surrey, UK) according to the manufacturer's protocol. Expression level of osteogenic genes was quantified by SYBR GreenER™ qPCR for iCycler kit (Invitrogen) using iCycler Thermal Cycler (Bio-Rad). The primer sequences used are: *OPN F* 5’-CTCCATTGACTCGAACGACTC-3’ *R* 5’-CAGGTCTGCGAAACTTCTTAGAT-3’; *BSP F* 5’-GAATGGCCTGTGCTTTCTCAA-3’ *R* 5’-TCGGATGAGTCACTACTGCCC-3’; *COLA1 F* 5’-AGACGAAGACATCCCACCAATC-3’ *R* 5’-AGATCACGTCATCGCACAACA-3’; *β-actin F* 5’-ATGTGGCCGAGGACTTTGATT -3’ *R* 5’-AGTGGGGTGGCTTTTAGGATG-3’. Transcript levels were normalized to the housekeeping gene β-actin and analyzed by the relative quantification 2^-ΔΔCt^ method.

### Western blot analysis

After hMSCs were cultured in osteogenic induction medium for up to 6 days, or transfected with miR-135b inhibitor or mimic for 3 days, cells were harvested, lysed, and protein extracts were blotted as previously described [20], using the antibodies of SMAD5 and β-actin (Both from Cell Signalling, Danvers, USA).

### Viability assays

hMSCs were cultured at a density of 2000 cells/well in a 96-well plate in the presence of lipofectamine, miR-135b inhibitor or mimic. After the indicated number of days, cell viability was measured with the use of CellTiter-Glo^®^ (Promega, Madison, USA) according to the manufacturer's protocol. 

### Osteogenic differentiation induction

Osteogenic differentiation was induced by culturing hMSCs at passage two in Osteogenic Induction Medium (Lonza,Verviers, Belgium). The medium was changed every 3 days. As a negative control, cells were cultured in complete growth medium and medium was changed at the same frequency as that for the differentiating MSCs. The Osteogenic Induction Medium contains according to the manufacturer dexamethasone, L-Glutamine, ascorbate, Pen/Strep, MCGS and glycerophosphate. Quantitative alkaline phosphatase (ALP) activity, qPCR for bone formation markers, and Alizarin Red S staining were used to evaluate osteogenic differentiation and performed at day 3, day 7 and day 14, respectively.

### Quantitative ALP activity measurement

2x10^3^ hMSCs at passage two were cultured with Osteogenesis Induction Medium (Lonza) in 96-well plate for 72 hours. The activity of ALP, an early osteogenic marker, was measured by quantitative ALP staining using alkaline phosphatase yellow (pNPP) liquid substrate system for ELISA (Sigma-Aldrich, Bornem, Belgium) at 415nm. To exclude the effect of difference in cell growth, ALP activity was normalized to total protein determined by the Pierce® BCA protein assay (Thermo Fisher Scientific, Epsom, UK) to determine alkaline phosphatase index (Alkaline phosphatase index = alkaline phosphatase spectrophotometer reading/protein× 1000).

### Qualitative ALP staining

2x10^4^ hMSCs were cultured with Osteogenesis Induction Medium (Lonza) in 24-well plate for indicated days. Qualitative ALP staining was performed by adding BCIP/NBT (5-Bromo-4-chloro-3-indolyl phosphate/Nitroblue tetrazolium, Sigma-Aldrich) liquid substrate to each well. ALP expression can be visualized in dark purple color.

### Alizarin Red S staining

Calcium deposits were evaluated as a late osteogenic marker by Alizarin Red S staining. 2x10^4^ hMSCs were cultured with Osteogenesis Induction Medium (Lonza) in a 24-well plate for two weeks. Cells were fixed with 10% paraformaldehyde (Merck) for 15 min and then stained with 40mM freshly Alizarin Red solution (pH=4.2, Sigma-Aldrich) for 10 min with gentle shaking followed by five washings (including washings with gentle shaking for 5min twice) with MilliQ water to reduce non-specific staining. Calcium deposits were visualized in red color.

### miR-135b gain and loss of function analysis

The miR-135b gain and loss of function analyses were performed by transfection of miR-135b inhibitor (MIN0000758, Qiagen) and miR-135b mimic (MSY0000758, Qiagen) together with Lipofectamine™ RNAiMAX Reagent (Invitrogen) according to the manufacturer's protocol. AllStars Negative Control siRNA (1027280, Qiagen) and miScript Inhibitor Negative Control (1027271, Qiagen) were used as negative controls for transfection of miR-135b mimic and miR-135b inhibitor, respectively, as the manufacturer suggested. Osteogenic medium with miR-135b mimic and inhibitor (and their controls) was replaced every three days.

### Indirect coculture

ND-hMSCs cultured in complete hMSC growth medium were pre-plated in a 6-well plate overnight and co-cultured using transwell systems (0.4μM pore size, 24mm insert, Corning, NY, USA) with human MM cells (RPMI8226 or U266) which were plated in the upper insert for 3 days or 6 days (hMSCs:MM cells=1:10), and further cultured for up to 14 days after removal of MM cells. hMSCs were harvested on the indicated day and the expression of miR-135b was tested. ND-hMSCs that were not co-cultured with MM cells, were harvested on the same day and used as negative control. 

### Statistical Analysis

Statistical analysis was done using GraphPad Prism 5 software. All data represent the mean ± SD. Results were analyzed using the Mann Whitney test (for two groups), or one way ANOVA followed by Tukey’s post test (for more than two groups), and Spearman’s correlation. *p*<0.05 was considered statistically significant. All experiments were repeated in at least triplicates.

## Results and Discussion

**Figure 1:**
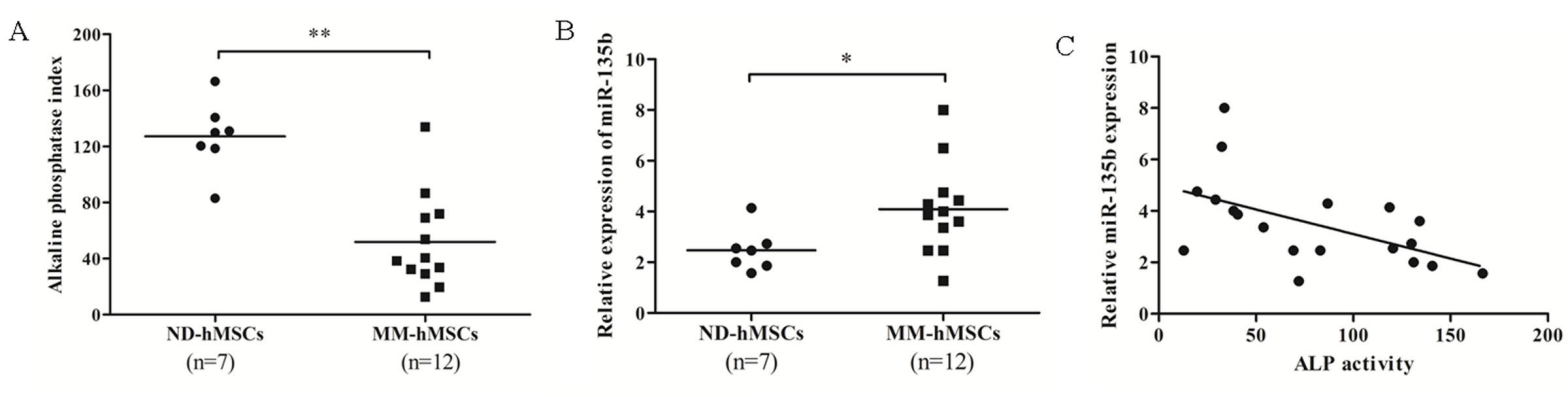
miR-135b expression is higher in MM BM-derived hMSCs showing impaired osteogenic differentiation potential. (A) MM-hMSCs show a reduced ALP activity, a marker for osteogenic differentiation. ND-hMSCs (n=7) and MM-hMSCs (n=12) are cultured in osteogenic medium (OM) for 72 hours and the ALP activity is measured quantitatively by the alkaline phosphatase yellow (pNPP) liquid substrate system for ELISA (Sigma-Aldrich, Bornem, Belgium). ** *p*<0.01 (B) miR-135b expression is significantly upregulated in MM-hMSCs compared to ND-hMSCs by quantitative real time PCR. The miR-135b expression of all hMSCs samples is normalized to the miR-135b expression of U266 MM cells. * *p*<0.05 (C) miR135b expression is inversely correlated with the ALP activity in MM-hMSCs. The relative expression of miR-135b in hMSCs derived from 12 MM patients was plotted versus their ALP activity.

**Figure 2:**
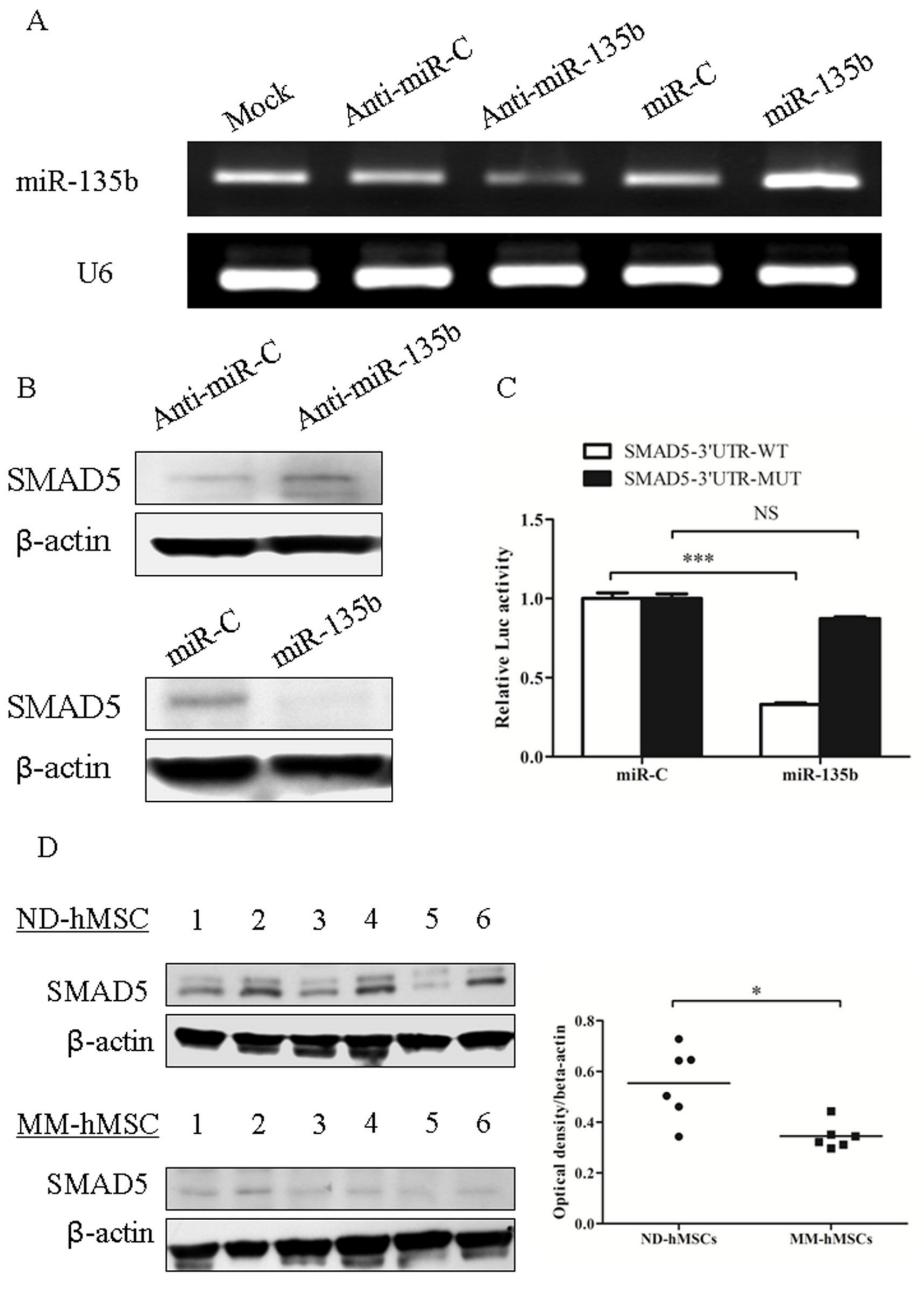
miR-135b negatively regulates SMAD5 expression. (A) Transfection with miR-135b inhibitor or mimic leads to a decrease or an increase of miR-135b expression in hMSCs. One of three independent experiments is shown. (B) miR-135b is confirmed to target SMAD5 in hMSCs. Transfection with miR-135b inhibitor or mimic leads to an increase or a decrease of SMAD5 expression, respectively, by Western blot in hMSCs. One of three independent experiments is shown. (C) HEK 293 cells were cotransfected with the luciferase reporters carrying wild-type or mutated SMAD5 3’UTR, and 50 nM miR-135b mimic or negative control (miR-C) for 48 h. The luminescence of Renilla luciferase was normalized to that of firefly luciferase, and the relative luminescence units was plotted. Normalized data are shown as mean±SD. n=3. ***, p<0.001; NS, not significant. (D) MM-hMSCs with upregulated miR-135b expression have a lower SMAD5 expression as shown by Western blot analysis. Left panels show the blots; right panels show densitometric analysis using ImageJ software. *p<0.05.

It has been described in several studies that miR-135b is abnormally expressed in some types of tumor and could contribute to tumorigenesis [21-28]. Importantly, miR-135b has also been reported to be involved in the regulation of osteogenic differentiation [15,29]. Using a high-throughput miRNA array, we found miR-135b to be one of the upregulated miRNAs in MSCs from MM patients as compared to those from normal donors. Because of its involvement in osteogenesis, this particular miRNA was selected for further study. Our previous data have demonstrated that MM-hMSCs have an impaired osteogenic differentiation ability compared to ND-hMSCs which is related to an abnormal deactivation of Notch signaling [5]. However, inhibition of Notch signaling could not completely restore MM-hMSCs osteogenic differentiation, indicating there might be other mechanisms involved. We hypothesized that abnormal expression of miR-135b in MM-hMSCs might be one possible mechanism. To test our hypothesis, we performed quantitative real time PCR (qPCR) to analyze miR-135b expression in 7 ND-hMSCs and 12 MM-hMSCs samples. The clinical characteristics of the MM patients included are shown in the table 1. We confirmed that MM-hMSCs have impaired osteogenic potential as shown by decreased alkaline phosphatase (ALP) activity (Figure 1A). Meanwhile, qPCR data demonstrated that miR-135b is significantly upregulated in MM-hMSCs in comparison to ND-hMSCs (Figure 1B). Noticeably, the relative expression of miR-135b in MM-hMSCs was inversely correlated to their ALP activity (R^2^= 0.3332, P<0.05, Figure 1C). It indicates that the upregulation of miR-135b in MM-hMSCs seems to be associated with their impaired osteogenic differentiation. Previous study has demonstrated by 3’UTR miRNA target validation that murine SMAD5 is one target of murine miR-135a (mmu-miR-135a) [29]. The sequence of miR-135a/b and 3’UTR of SMAD5 are both highly conserved among the different species (Figure S1). So it is expected that miR-135b can also target SMAD5 in human MSCs. We transfected hMSCs with miR-135b inhibitor or mimic, and verified the efficacy of transfection by PCR (Figure 2A). As expected, we observed by Western blot that miR-135b inhibitor or mimic increased or decreased SMAD5 expression in hMSCs, respectively (Figure 2B). Moreover, to test this prediction, wild-type or mutated putative 3’UTR coding sequences for SMAD5-binding sites are cloned into a luciferase reporter plasmid. Functional luciferase activity assay showed that ectopic expression of miR-135b significantly inhibited the luciferase activity of wild-type Smad5 3’UTR reporter plasmids, but had no effect on mutated Smad5 3’UTR reporters (Figure 2C). These results suggest that miR-135b binds directly to the predicted binding sites in the SMAD5 3’-UTR and negatively regulates SMAD5 expression. Furthermore, we selected 6 ND-hMSCs and 6 MM-hMSCs with high miR-135b expression and determined by Western blotting that the baseline expression of SMAD5 in MM-hMSCs was generally lower compared to ND-hMSCs (Figure 2D), in accordance with their differential miR-135b expression. However, we admit that besides SMAD5, there are other targets of miR-135b which are involved in MSC osteogenesis [30]. This deserves our further investigation. 

**Table 1:** The clinical characteristics of the MM patients.

Patient No.	Age	Gender	Disease classification	Relapse	Bone lesion
1	87	Male	Active	No	Yes
2	75	Female	Inactive	No	No
3	49	Male	Active	Yes	No
4	61	Female	Active	No	Yes
5	70	Male	Active	Yes	Yes
6	77	Female	Active	Yes	Yes
7	69	Female	Active	Yes	Yes
8	48	Female	Active	No	Yes
9	63	Male	Active	Yes	Yes
10	78	Male	Active	No	Yes
11	51	Female	Active	No	Yes
12	75	Male	Active	Yes	Yes

**Figure 3:**
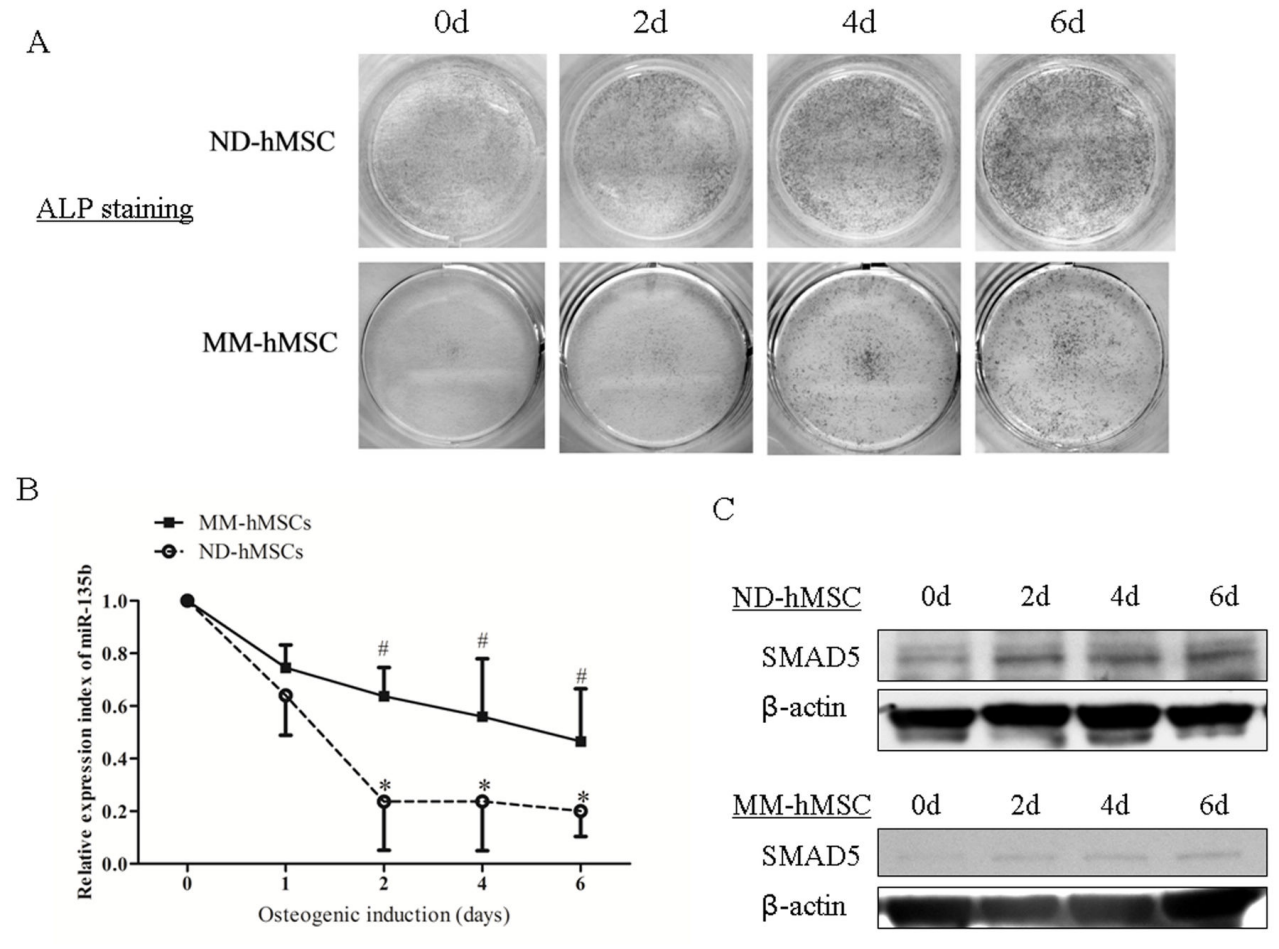
MM-hMSCs exhibit a different miR-135b expression during osteogenic differentiation compared to ND-hMSCs. (A) The osteogenic differentiation of ND-hMSCs and MM-hMSCs gradually progressed when exposed to osteogenic induction medium (OM) *in* *vitro* as shown by qualitative ALP staining. However, the ALP increase in MM-hMSCs is lower compared to ND-hMSCs. (B) miR-135b relative expression, as detected by quantitative real time PCR, decreases during osteogenesis significantly in both ND-hMSCs and MM-hMSCs. There is a delay of miR-135b decrease in MM-hMSCs. n=5/group. * and # indicate *p*<0.05, compared to day 0 for ND-hMSCs and MM-hMSCs, respectively. All values are normalized to day 0. (C) MM-hMSCs with impaired osteogenic differentiation show a less increasing level of SMAD5 during osteogenic differentiation compared to ND-hMSCs. One representative result of three is shown.

Having confirmed that the expression of miR-135b was upregulated in MM-hMSCs, we studied further its expression pattern during hMSCs osteogenesis. ND-hMSCs and MM-hMSCs were cultured in osteogenic inductive conditions and harvested at different time points. ALP staining was performed in order to verify the osteogenic differentiation (Fig. 3A). Using qPCR we observed a considerable decrease of miR-135b expression in both ND-hMSCs and MM-hMSCs after osteogenic induction. However, there was a delay of miR-135b decrease in MM-hMSCs (Figure 3B). Interestingly, we observed by Western blotting that SMAD5 increased during osteogenic differentiation in both ND-hMSCs and MM-hMSCs. However, the increase in MM-hMSCs was lower as compared to ND-hMSCs (Figure 3C). 

**Figure 4:**
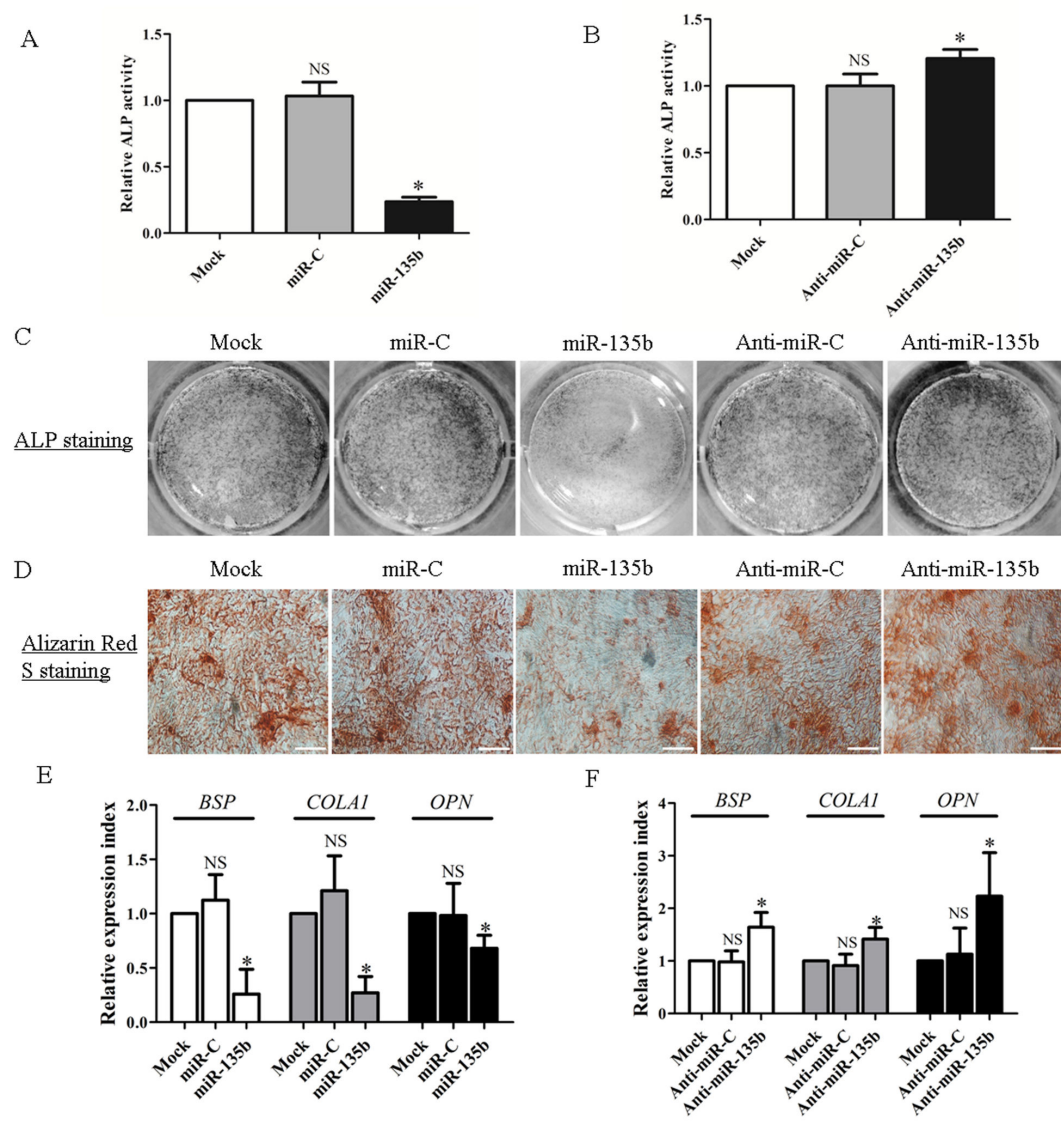
miR-135b negatively regulates the hMSC osteogenic differentiation. (A) ND-hMSCs are transfected with miR-135b mimic (50nM), miRNA mimic negative control (miR-C) (50nM) or transfection reagent only (Mock) within OM for 3 days. ALP activity is downregulated significantly in miR-135b overexpressing ND-hMSCs. n=4. (B) ND-hMSCs are transfected with miR-135b inhibitor (Anti-miR-135b) (50nM), miRNA inhibitor negative control (Anti-miR-C) (50nM) or transfection reagent only (Mock) within OM for 3 days. ALP activity is upregulated significantly in miR-135b knockdown ND-hMSCs. n=4. (C) ND-hMSCs are cultured in OM with miR135b mimic, miRNA mimic negative control, miR135b inhibitor, miRNA inhibitor negative control or transfection reagent only for 6 days. ALP staining is performed using BCIP/NBT solution and confirms that miR-135b gain or loss results in a decrease or increase of ALP expression, respectively. One representative result of three is shown. (D) Alizarin Red S staining performed at day 14 shows a significant reduction of mineralization after miR135b mimic transfection and an enhanced mineralization after miR135b inhibitor transfection. Original magnification: ×100. Scale bar, 200μm. One representative result of three is shown. (E) ND-hMSCs are cultured in OM with miR135b mimic, miRNA mimic negative control, or transfection reagent only for one week. Bone formation markers (*BSP*, *COLA1* and *OPN*) show a significant decrease in miR-135b overexpressing ND-hMSCs by quantitative real time PCR. n=3. (F) ND-hMSCs are cultured in OM with miR135b inhibitor, miRNA inhibitor negative control, or transfection reagent only for one week. Bone formation markers (*BSP*, *COLA1* and *OPN*) show a significant increase in miR-135b knockdown ND-hMSCs. n=3. * *p*<0.05; NS, not significant, compared to mock group. All values are normalized to the value obtained with mock. Abbreviations: ALP, alkaline phosphatase; OPN, osteopontin; BSP, bone sialoprotein; COLA1, collagen type І.

To verify further whether miR-135b is functionally involved in the osteogenic commitment of BM derived hMSCs, ND-hMSCs were transfected with miR-135b inhibitor and miR-135b mimic in osteogenic medium. Transfection reagent alone (mock) did not influence cell viability (Figure S2A) or osteogenic differentiation (Figure S2B) significantly and was used as control. We found that transfection with miR-135b inhibitor and miR-135b mimic induced, respectively, a gain and loss of function in ND-hMSCs osteogenesis. However, we did not observe any effect on hMSC viability when cells were incubated with miR-135b inhibitor and mimic (Figure S3). In fact, ND-hMSCs, transfected with miR-135b mimic, exhibit a very significant decrease of ALP activity compared to the mock or mimic negative control transfected cells (Figure 4A), whereas transfection with miR-135b inhibitor shows a higher level of ALP activity compared to the controls (Figure 4B), and this is confirmed by ALP staining (Figure 4C) and mineralization staining (Figure 4D). In addition, qPCR results showed a major expression drop of the osteogenic markers (*BSP*, *COLA1*, *OPN*) upon miR-135b mimic transfection (Figure 4E) and a significant upregulation after miR-135b inhibitor transfection (Figure 4F). In addition, we observed that Notch signaling inhibition by DAPT could not influenced significantly miR-135b and SMAD5 expression, and Notch signaling downstream genes were not affected significantly by miR-135b either, indicating that there was no link between Notch signaling and miR-135b/SMAD5 in MSC osteogenesis (Figure S4).

**Figure 5:**
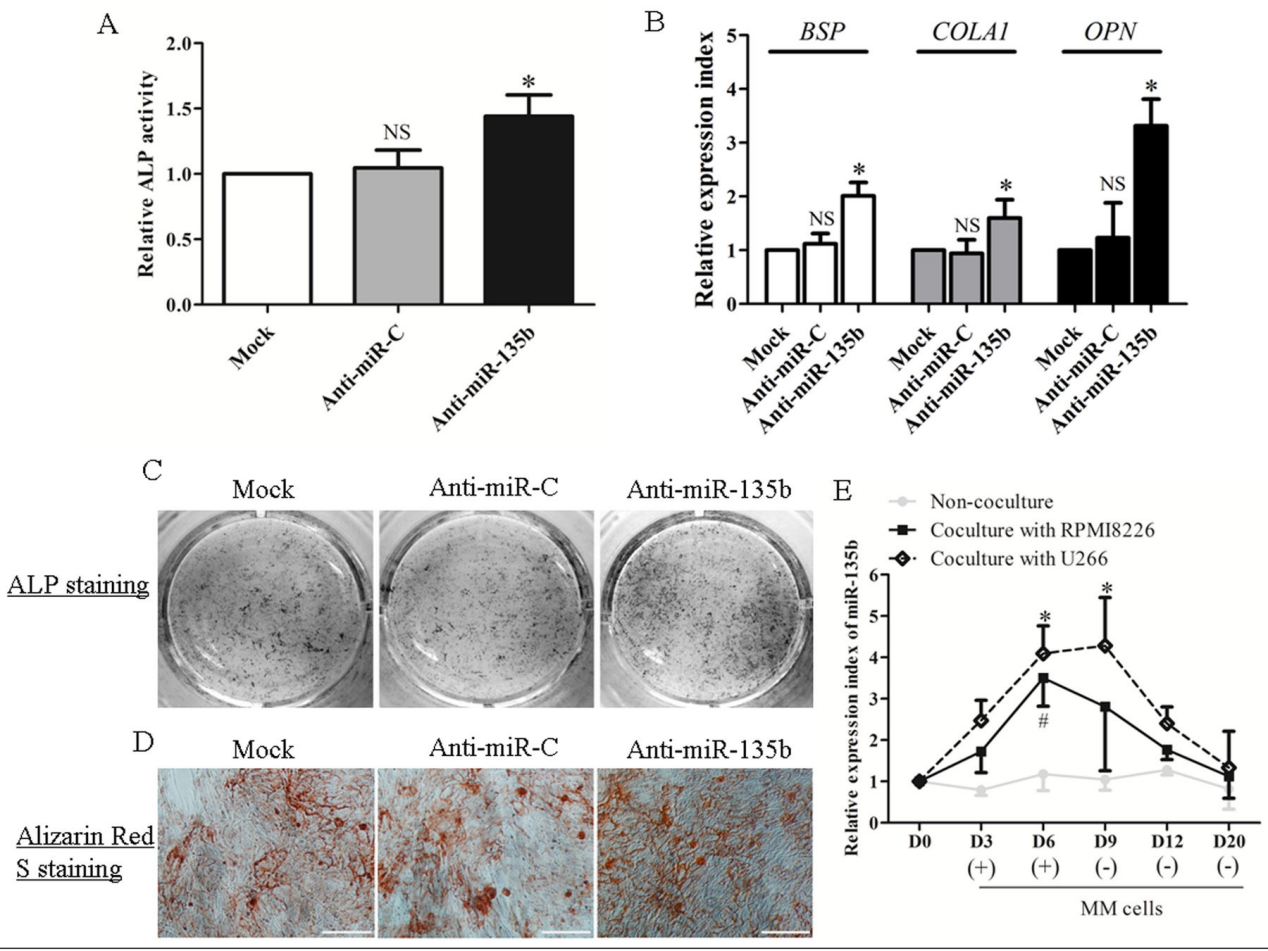
Inhibition of miR-135b has a therapeutic effect to restore the impaired osteogenic potential of MM-hMSCs. (A) Transfection of miR-135b inhibitor (50nM) in osteogenic induction medium (OM) for 3 days significantly increases ALP activity for MM-hMSCs. n=4. * *p*<0.05; NS, not significant, compared to mock group. (B) MM-hMSCs are cultured in OM supplemented with miR-135b inhibitor (50nM), miRNA inhibitor negative control or transfection reagent only for one week. Bone formation markers (*BSP*, *COLA1* and *OPN*) show a significant increase in miR-135b knockdown MM-hMSCs by quantitative real time PCR. n=3. * *p*<0.05; NS, not significant, compared to mock group. (C) ALP staining confirms that loss of miR-135b results in an increase in MM-hMSCs osteogenic differentiation. One representative result of three is shown. (D) Alizarin Red S staining also confirms that MM-hMSCs transfected with miR-135b inhibitor demonstrated an increased calcium deposit. Original magnification, ×100. Scale bar, 200μm. One representative result of three is shown. (E) Upregulation of miR-135b in ND-hMSCs is induced by coculture of MM cells RPMI8226 and U266, and gradually returns to normal level after removal of MM cells. n=3. * and # indicate *p*<0.05, compared to day 0 for U266 and RPMI8226 coculture, respectively. ND-hMSCs without MM cell coculture were used to exclude the possible influence of cell confluence on miR-135b expression.

Given the osteogenesis-supporting effects obtained in ND-hMSCs, we also validated the therapeutic effect of miR-135b inhibitor on the improvement of osteogenic differentiation in MM-hMSCs (Figure 5A-5D). In a previous study we demonstrated that an abnormal cytokine expression can be induced in MSCs by co-culture with MM cells [31]. Therefore we were also interested to investigate whether MM cells can induce an abnormal expression of miR-135b. By co-culturing ND-hMSCs with MM cells (RPMI8226 or U266) for 3 and 6 days, we observed a significant upregulation of miR-135b expression (Figure 5E). After removing the MM cell exposure, the upregulated miR1-35b expression returned almost to the normal level. This seems to contrast with our observation that MM-hMSCs maintain an upregulated miR-135b expression even though the cells have been isolated from the MM tumor microenvironment and were expanded in vitro for a few weeks. However, during the long-term exposure to the MM tumor microenvironment in vivo, the mRNA and/or miRNA profile of MSCs might have undergone irreversible changes. Our data suggest that MM cell-produced soluble factors are involved in stimulating the upregulation of miR-135b in hMSCs. However, further studies are needed to identify these molecules.

Taken together, our results illustrate, for the first time, that MSCs derived from the malignant MM microenvironment show an enhanced miR-135b expression which is associated with their reduced osteogenic potential. Although several strategies have been explored for increasing the MSC osteoblastogenic capacity and treatment of MM bone disease, none of those could totally correct MM cell induced bone lesions, indicating that new treatment modalities are necessary. Targeting MM-hMSCs by miR-135b inhibitors might be an alternative strategy to control the impaired MSC osteogenic differentiation and to stimulate bone formation.

## Supporting Information

Figure S1 **The sequence of miR-135a/b and 3’UTR of SMAD5 are both very conservative among different species.** (A) The sequence of miR-135 which targets SMAD5 (AUGGCUU, as underlined) is very conservative. There is only one nucleotide difference between miR-135a and miR-135b (in red). (B) The sequence in the 3’UTR of SMAD5 which is regulated by miR-135 (AAGCCAU, as underlined) is also very conservative (From www.targetscan.org and www. microrna.org).
(TIF)

Figure S2 **Transfection reagent does not influence the viability and osteogenic differentiation of hMSCs in vitro.** (A) hMSCs are cultured in growth medium with (mock) or without (non-treated) lipofectamine for 3 days and 7 days. The viability of hMSCs is not affected significantly by lipofectamine. (B) hMSCs are cultured in osteogenic induction medium with (mock) or without (non-treated) lipofectamine for 7 days. The expression for osteogenic markers of hMSCs is also not affected significantly by lipofectamine. n=3. NS: not significant, compared to non- treated group.
(TIF)

Figure S3 **Transfection of miR-135b inhibitor and mimic does not influence the viability of hMSCs in vitro.** hMSCs are cultured in growth medium with lipofectamine (mock), miR-135b inhibitor and mimic for 3 days and 7 days. The viability of hMSCs is not affected significantly by miR-135b inhibitor or mimic transfection. n=3 NS: not significant, compared to mock group.
(TIF)

Figure S4 **No link between miR135b/SMAD5 and Notch signaling.** (A) Treatment of Notch signaling inhibitor DAPT at 10μM for 1d and 3d did not change significantly the miR135b (left panel) or SMAD5 (right panel) expression in MSCs by qPCR. (B) Treatment of miR-135b inhibitor did not change significantly the expression of Notch signaling downstream genes hes1, hes5, hey1 and hey2 by qPCR. (C) miR-135b/SMAD5 induced osteogenesis was not through Notch signaling, and vice versa.
(TIF)
