# Supplementary figures and images for: Upregulation of miR-135b Is Involved in the Impaired Osteogenic Differentiation of Mesenchymal Stem Cells Derived from Multiple Myeloma Patients
Source: PLoS One. 2013 Nov 6;8(11):e79752. doi: 10.1371/journal.pone.0079752 (PMC3819242; doi:10.1371/journal.pone.0079752)

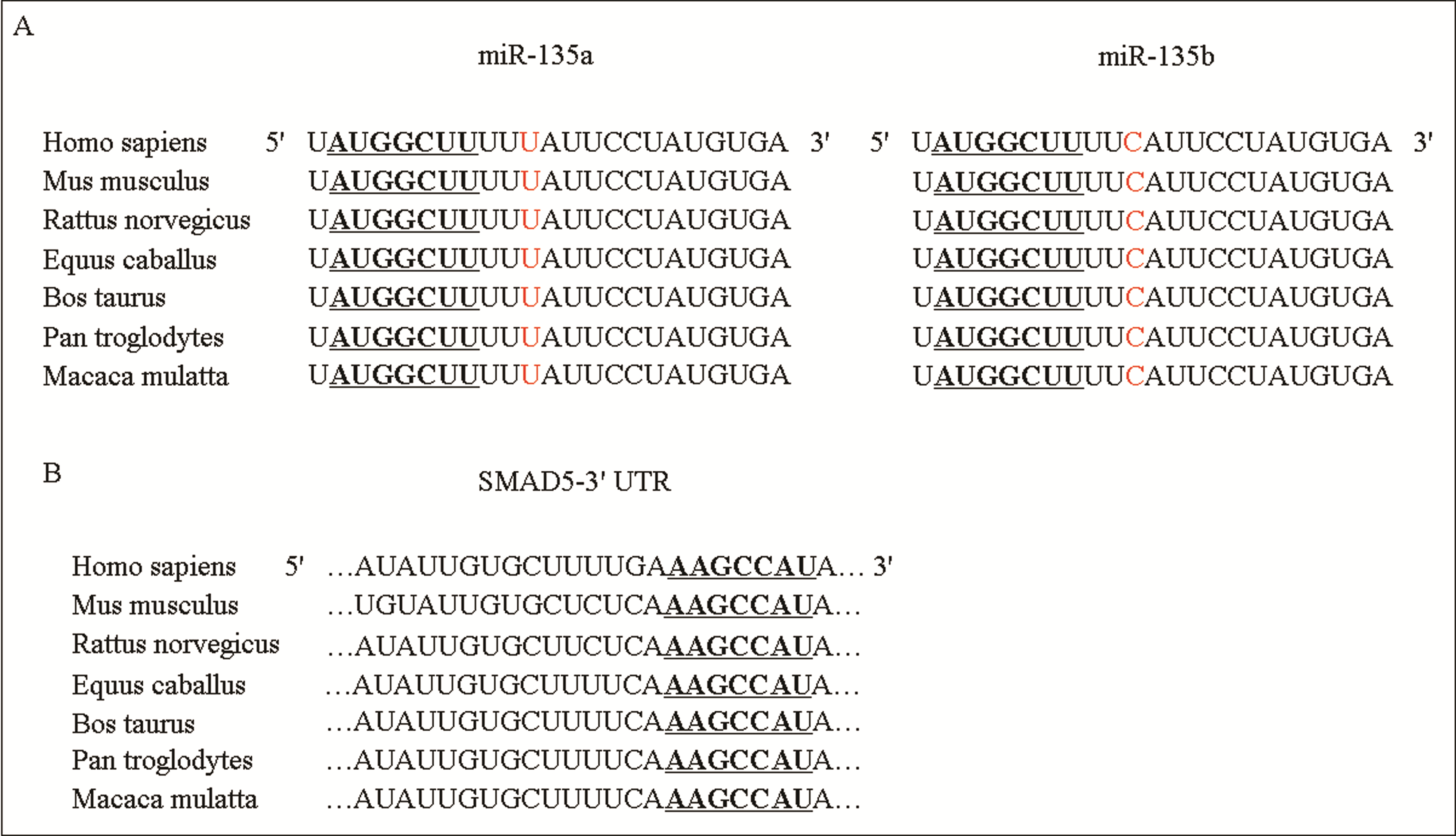

Supplement: Figure S1 — The sequence of miR-135a/b and 3’UTR of SMAD5 are both very conservative among different species. (A) The sequence of miR-135 which targets SMAD5 (AUGGCUU, as underlined) is very conservative. There is only one nucleotide difference between miR-135a and miR-135b (in red). (B) The sequence in the 3’UTR of SMAD5 which is regulated by miR-135 (AAGCCAU, as underlined) is also very conservative (From www.targetscan.org and www. microrna.org). (TIF) [file pone.0079752.s001.tif]

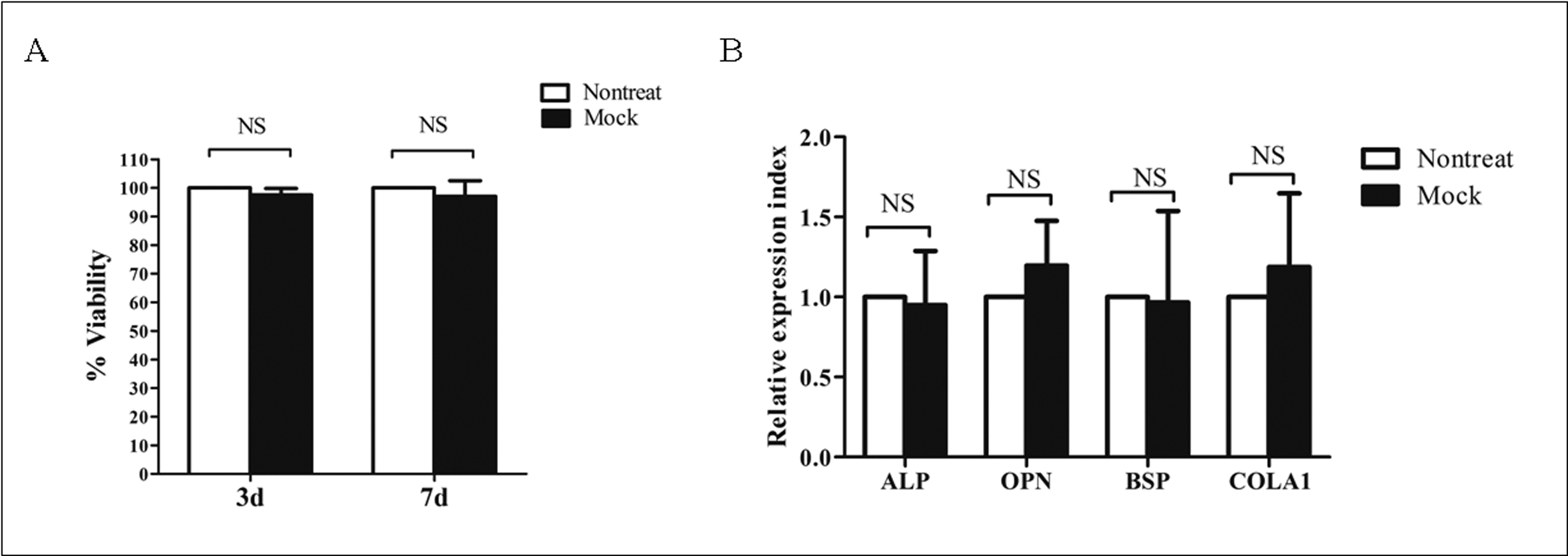

Supplement: Figure S2 — Transfection reagent does not influence the viability and osteogenic differentiation of hMSCs in vitro. (A) hMSCs are cultured in growth medium with (mock) or without (non-treated) lipofectamine for 3 days and 7 days. The viability of hMSCs is not affected significantly by lipofectamine. (B) hMSCs are cultured in osteogenic induction medium with (mock) or without (non-treated) lipofectamine for 7 days. The expression for osteogenic markers of hMSCs is also not affected significantly by lipofectamine. n=3. NS: not significant, compared to non- treated group. (TIF) [file pone.0079752.s002.tif]

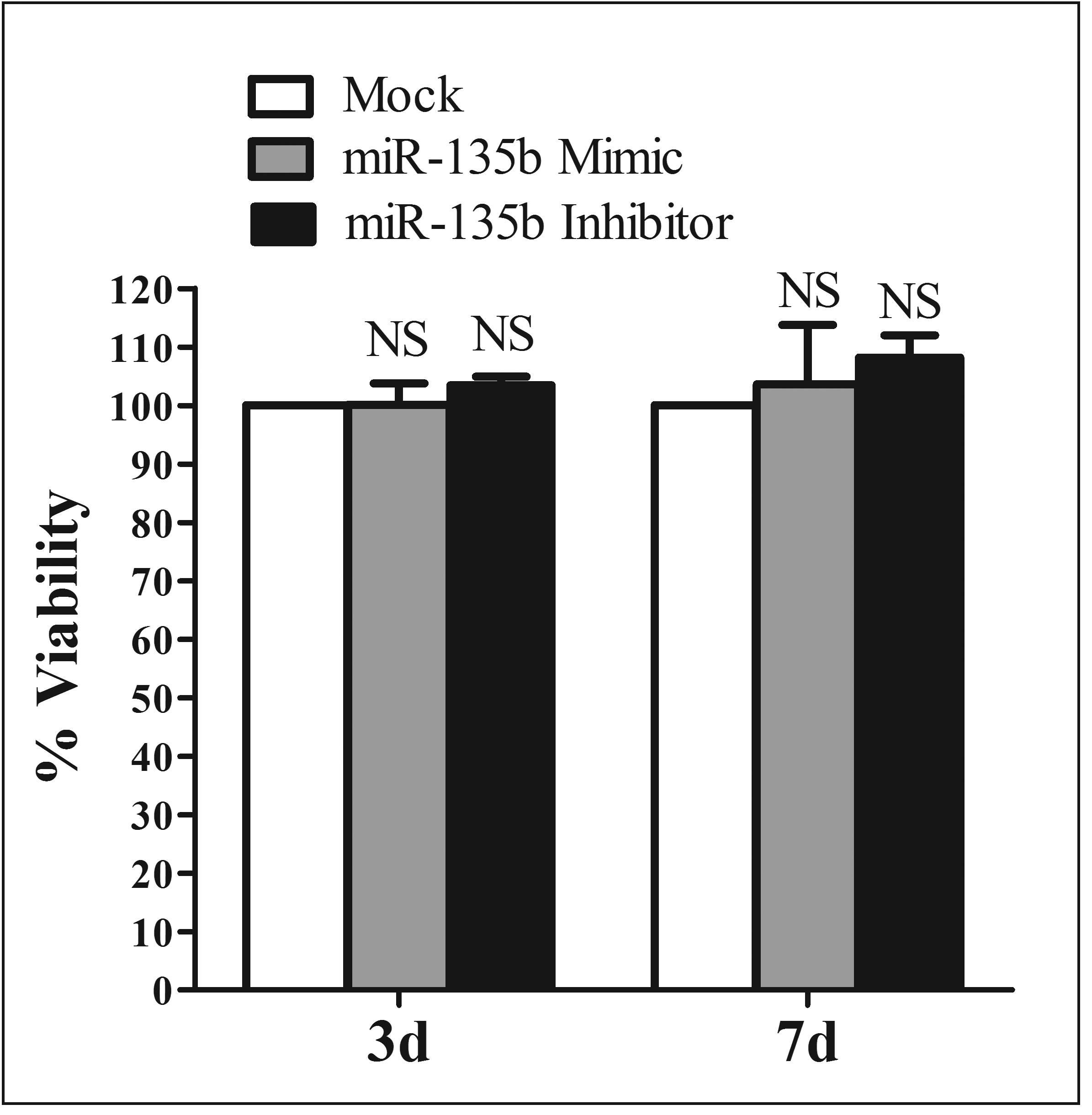

Supplement: Figure S3 — Transfection of miR-135b inhibitor and mimic does not influence the viability of hMSCs in vitro. hMSCs are cultured in growth medium with lipofectamine (mock), miR-135b inhibitor and mimic for 3 days and 7 days. The viability of hMSCs is not affected significantly by miR-135b inhibitor or mimic transfection. n=3 NS: not significant, compared to mock group. (TIF) [file pone.0079752.s003.tif]
